# Dietary Vitamin A Intake and Associated Factors Among Lactating Women in Wondo Genet District, Sidama Region, Southern Ethiopia

**DOI:** 10.1155/jnme/5560305

**Published:** 2026-02-02

**Authors:** Amelo Bolka, Selamawit Sikuare, Aregahegn Dona, Assefa Philipos

**Affiliations:** ^1^ School of Public Health, Yirgalem Hospital Medical College, Yirgalem, Ethiopia

**Keywords:** associated factors, lactating women, Sidama Region, vitamin A adequacy, vitamin A intake

## Abstract

**Background:**

Vitamin A deficiency remains a public health problem in Ethiopia, despite programs aimed at providing lactating women with diverse diets and micronutrient supplements. However, evidence on the dietary vitamin A intake adequacy among these is limited. This study was aimed at assessing dietary vitamin A intake and associated factors among lactating women in the Wondo Genet district of Sidama Region, Southern Ethiopia.

**Methods:**

A community‐based cross‐sectional study was conducted among 411 lactating women from January 1 to 28, 2024, using simple random sampling. Data were collected via an interviewer‐administered questionnaire. Vitamin A inadequacy was defined as dietary intake below the estimated average requirement (EAR). Logistic regression was used to identify factors associated with dietary vitamin A inadequacy, and results are presented as adjusted odds ratios (AORs) with 95% confidence intervals (CIs).

**Results:**

The magnitude of inadequate dietary Vitamin A intake was 47.1% (95% CI: 42.2%, 52.0%). In the adjusted model, large family size (AOR = 1.68, 95% CI: 1.10, 2.58), food insecurity (AOR = 1.60, 95% CI: 1.05, 2.44), low dietary diversity (AOR = 1.65, 95% CI: 1.05, 2.62), not receiving nutritional counseling (AOR = 2.67, 95% CI: 1.66, 4.28), and consuming fewer than three meals per day (AOR = 2.18, 95% CI: 1.41, 3.37) were significantly associated with inadequate vitamin A intake.

**Conclusion:**

The study found a high prevalence of inadequate dietary vitamin A intake among lactating women in the area. Predictors of inadequate intake included large family size, food insecurity, lack of nutritional counseling, low dietary diversity, and the number of meals consumed per day. We recommend targeted nutritional counseling and education for lactating women, emphasizing vitamin A awareness, meal planning, and strategies to combat food insecurity, particularly in larger families.

## 1. Background

Vitamin A is an essential nutrient required by the body in minimal quantities for proper functioning and overall health [1]. It is a fat‐soluble nutrient primarily obtained from dietary sources such as organ meats, milk, and carotenoids found in vegetables and fruits [2]. The diet provides two forms of vitamin A: preformed vitamin A from animal sources and provitamin A carotenoids from plant sources [3, 4]. Adequate intake of this nutrient supports both maternal well‐being and the development of infants, contributing to immune function, vision, and cellular growth [5]. Lactating women experience heightened nutritional demands during the postpartum period, making adequate vitamin A intake essential for supporting maternal health and ensuring sufficient transfer to breast milk for their infants [6].

Despite its importance, lactating women in low‐ and middle‐income countries face challenges in achieving adequate vitamin A intake [7]. Multiple factors influence vitamin A intake among them, including socioeconomic status, education, dietary practices, cultural beliefs, and access to healthcare services [3]. Recognizing this critical issue, the World Health Organization (WHO) emphasizes the urgent need to promote adequate dietary vitamin A intake among lactating women [1].

Vitamin A deficiency in lactating women can arise from inadequate intake of vitamin A‐rich foods or digestive problems that hinder absorption [7]. This deficiency poses serious health risks, including weakened immune function, poor reproductive health, increased risk of anemia, and potential vision problems [1, 8]. It not only impacts maternal health but also reduces the quality of breast milk, adversely affecting infant growth, development, and immunity. As a result, both mothers and infants face higher risks of morbidity and mortality [9].

Vitamin A deficiency poses a significant public health challenge in low‐ and middle‐income countries, with the highest prevalence observed in sub‐Saharan Africa and South Asia [7]. In Ethiopia, studies have revealed that vitamin A deficiency has been a public health concern [10, 11]. The Ethiopian national micronutrient survey revealed a 3.4% prevalence of vitamin A deficiency among women of reproductive age [12].

The Ethiopian government, in collaboration with stakeholders, is implementing a Revised National Nutrition Program to ensure lactating women have access to diversified diet and micronutrient supplements. Strategies included promoting home gardening, community horticulture, fruit and vegetable consumption, urban gardening, access to animal source foods, and conducting public awareness campaigns about the health benefits of vitamin A [13]. Despite these efforts, studies on vitamin A intake among lactating women in the study area and the region are lacking. This study aimed to assess dietary vitamin A intake and associated factors among lactating women in the Wondo Genet district, Sidama Region, Southern Ethiopia.

## 2. Methods

### 2.1. Study Period, Design, and Setting

From January 1 to 28, 2024, a community‐based cross‐sectional study on lactating women was conducted in Wondo Genet district, Sidama Region. Located 24 km east of Hawassa City and 270 km south of Addis Ababa, the district spans altitudes of 1761–2695 m above sea level and includes 62% midlands and 38% highlands. Over 85% of the population relies on agriculture for their livelihood, cultivating key crops such as enset, maize, wheat, barley, teff, various fruits, vegetables, and root crops, while khat functions as a cash crop. The projected population for the district in 2024 was 160,536, which encompasses 5958 lactating women. The area has a population density of 768 people per square kilometer and is served by 1 district hospital, 3 health centers, and 13 operational health posts [14].

### 2.2. Study Population and Their Eligibility Criteria

Lactating women who lived in the study area for at least six months during the study period were eligible to take part in the study. Those who were too ill or mentally unstable to respond were excluded.

### 2.3. Sample Size Determination and Sampling Procedure

The required sample size for assessing the prevalence of inadequate vitamin A intake was calculated using the single population proportion formula. The calculation was based on the following parameters: a 95% confidence level, 80% power, 58.2% prevalence of inadequate dietary Vitamin A intake [15], and 10% allowance for the potential nonresponse. This process yielded a final required sample size of 411 participants.

From the 15 kebeles within the district, 6 were chosen by simple random sampling. A sampling frame was constructed using health post registries of lactating mothers residing in the selected kebeles. The total sample size was allocated proportionally to each kebele according to its population of lactating mothers. Finally, individual study participants were selected from each kebele using simple random sampling.

### 2.4. Data Collection Instruments and Procedures

A standardized, pretested, interviewer‐administered questionnaire was used for data collection. The tool was developed in English based on an extensive review of the literature and then translated into the local language, Sidamu Afoo, for field implementation. It was designed to address the study objectives and adapted from previously published studies [15–18].

Data were collected through face‐to‐face interviews conducted at participants’ homes by four bachelor’s‐level health officers using structured questionnaires. The overall data collection process was supervised by the principal investigator and a supervisor with a Master’s degree in General Public Health. Twenty‐four‐hour dietary recall data were collected using a paper‐based format, whereas all other data were gathered using Kobo Toolbox.

### 2.5. Dietary Intake Assessment

To determine the vitamin A intake of lactating women, dietary data were collected using a multiple‐pass 24 h dietary recall method, previously adapted and validated for developing countries [19]. This four‐step method involved participants recalling 24 h food and beverage consumption (including time and location), providing detailed descriptions (ingredients and preparation), and reporting standardized portion sizes. Finally, the data were reviewed for completeness, accuracy, and error identification.

Prior to actual data collection, a preliminary assessment was conducted in the study area. This involved a market inspection and a survey of 5% of households to identify commonly consumed foods, prevalent cooking methods, and the types of utensils used. Photographs were taken of standard utensils and typical single‐meal food portions. Identified utensils were then purchased locally and assigned unique identification codes.

For consistent data collection, all utensils and food portions were systematically photographed and precisely measured using a digital food weighing scale and a measuring cylinder for liquids. Measurements were recorded in milliliters and grams, with a direct conversion of 100 mL to 100 g applied to beverages. These photographic records served as visual aids, assisting participants in accurately recalling and estimating the portion sizes of items they had consumed.

Following the dietary recall, participants were prompted with a precompiled list of staple foods relevant to the study period. Food quantities were then estimated using common household measures, classified into large, medium, or small categories based on the amount consumed. Participants also utilized a photographic atlas to identify specific utensils and estimated portion sizes with the aid of provided measurement equipment. For commercially purchased foods, brand names and quantities were documented, and these items were subsequently acquired from the market to ascertain their vitamin A content from product labels. The vitamin A content of mixed dishes was determined through calculations based on their respective recipes.

Dietary data collection was intentionally avoided on fasting days and public holidays. To account for potential day‐to‐day variability in vitamin A consumption, a subset of lactating women (15%, *n* = 61) underwent repeated dietary data collection on nonconsecutive days, while the remaining participants completed a single 24 h recall. A statistical analysis revealed no significant difference in vitamin A intake between the initial and subsequent recall days (*p* > 0.05).

Dietary diversity was evaluated using an updated 24 h recall questionnaire developed by the Food and Agriculture Organization (FAO). This list‐based questionnaire prompted respondents to report their food intake from the preceding day, encompassing consumption both at home and elsewhere. The Women’s Dietary Diversity Score (WDDS) was then calculated by tallying the number of distinct food groups consumed from 10 predefined categories: starchy staples, pulses (e.g., beans, peas, lentils), nuts and seeds, dairy products, flesh foods, eggs, dark green leafy vegetables, vitamin A‐rich fruits and vegetables, other fruits, and other vegetables [20].

### 2.6. Household Food Security

Lactating women’s food security was measured using the Household Food Insecurity Access Scale (HFIAS). Participants were asked whether, in the last 4 weeks, they had (1) worried that their household would not have enough food, (2) been unable to eat the kinds of foods they preferred due to food shortages, (3) experienced limited variety in their diet because of a lack of food or money to buy food, (4) eaten foods they did not want to eat, (5) consumed smaller meals than they felt they needed, (6) had fewer meals in a day because there was not enough food or money, (7) gone without any food in their household due to a lack of resources, (8) gone to bed hungry at night because there was not enough food, and (9) gone a whole day and night without eating anything because there was not enough food. We coded “Yes” responses as 1 and “No” responses as 0, then summed the scores. Participants with a food insecurity score of 1 or higher were categorized as food insecure [21].

### 2.7. Nutritional Knowledge of Lactating Women

Lactating women’s vitamin A knowledge was evaluated with 18 questions covering four areas: foods rich in vitamin A, causes of deficiencies, consequences, and prevention methods. Each correct response earned one point, with incorrect answers getting zero. Finally, a cumulative score was computed [22, 23].

### 2.8. Variables of Study

The outcome variable of interest was dietary vitamin intake adequacy. On other hand, 11 independent variables were considered for the study. These were (1) husband education status, (2) husband occupation, (3) family size, (4) household food security, (5) place of delivery, (6) postnatal care, (7) nutrition education, (8) vitamin A knowledge, (9) dietary diversity, and (10) the number of meals consumed per day.

Dietary inadequacy was defined as an intake of vitamin A below the EAR of 900 μg RAE/day for lactating women; otherwise, it is considered adequate intake. Based on the recommended dietary allowance (RDA), inadequacy was defined as dietary vitamin A intake of less than 1300 μg RAE/day [24].

Lactating women who consumed five or more food groups were considered to have a high dietary diversity score. This score was calculated by summing the number of distinct food groups consumed within a 24 h recall period. Scores below five indicated a lower level of dietary diversity [25].

Lactating women’s knowledge on vitamin A: knowledge was considered good for scores at or above the mean and poor for scores below the mean [22, 23].

### 2.9. Data Management and Analysis

Data were collected using the Kobo Toolbox system and exported to SPSS version 25.0 for data management and analysis. Data were described using frequency distributions, measures of central tendency, and dispersion. Portion sizes obtained from 24 h dietary recalls were manually converted into grams. Nutritional values per 100 g were determined primarily using the Ethiopian Food Composition Tables (EFCTs); for food items not listed in the EFCT, food composition data from relevant African countries were utilized [26, 27]. The adequacy of lactating women’s dietary vitamin A intake was assessed by comparing their daily nutrient intake to the estimated mean requirement (EAR), with intake below the EAR indicating inadequacy. Dietary diversity was measured using the FAO standardized tool for women’s dietary diversity [28].

Bivariable and multivariable logistic regression analyses assessed the association between independent variables and the outcome. Variables with a *p* value < 0.25 from the bivariable model were included in the multivariable model to address confounding. Model fitness was evaluated using the Hosmer and Lemeshow tests (*p* = 0.958), and multicollinearity was checked with the variance inflation factor (VIF). Statistical significance was set at *p* < 0.05, with results presented as adjusted odds ratios (AORs) and 95% confidence intervals (CIs).

## 3. Results

### 3.1. Sociodemographic Characteristics of Respondent

The study included 401 respondents, yielding a response rate of 97.6%. The mean ( ± SD) age of the respondents was 26.8 ± 4.9 years. Most (78.8%) were of Sidama ethnicity, and the vast majority (87.3%) were protestant religion followers. In terms of education, 34.9% of women and 29.9% of their husbands had no formal schooling. Most participants were housewives (80.5%), while nearly half of their husbands were farmers (48.4%).

The respondents reported a median (interquartile range) monthly income of 2500 (IQR: 600‐5995) Ethiopian Birr (10.7–107.1 $US). The mean household size was 4.8 (±1.8) persons. A large majority of respondents (98%) owned farmland, and among these, 94.2% cultivated *Chat* as a cash crop. Of the farmland owners, 84.7% grew cereals such as maize, barley, and wheat, whereas 77.9% cultivated root and tuber crops including potatoes, sweet potatoes, enset, turnip, and onion. Slightly more than half of the respondents (58.8%) also cultivated fruits and vegetables—such as carrots, papaya, tomatoes, kale, cabbage, and pepper—on their farmland. Food insecurity was reported in 48.6% of households (Table 1).

**TABLE 1:** Sociodemographic/economic characteristics of lactating women in Wondo Genet district, Sidama Region.

Variable	Category	Frequency	Percent (%)
Ethnicity	Sidama	316	78.8
	Others	85	21.2

Religion	Protestant	350	87.3
	Others	51	12.7

Women education	Not attended formal education	140	34.9
	Primary education and above	261	65.1

Husband education	Not attended formal education	120	29.9
	Primary education and above	281	70.1

Women occupation	Housewife	323	80.5
	Other works	78	19.5

Husband occupation	Farmer	194	48.4
	Other works	207	51.6

Household monthly income	≤ 2500 Eth Birr (≤ 44.6 $US)	220	54.9
	> 2500 Eth Birr (> 44.6 $US)	181	54.1

Family size	< 5 members	214	53.4
	≥ 5 members	187	46.6

Owned farmland	Yes	393	98
	No	8	2

Use Khat as a cash crop	Yes	378	94.3
	No	23	5.7

Cultivate cereals on their farm land	Yes	333	84.7
	No	60	15.3

Cultivate roots and tubers on their farm land	Yes	306	77.9
	No	87	22.1

Cultivate fruits and vegetables on their farm land	Yes	231	58.8
	No	162	41.2

Food security	Secured	206	51.4
	Insecure	195	48.6

### 3.2. Health Service Utilization of the Lactating Women

Maternal healthcare service utilization is detailed in Table 2. Most lactating women (80.8%) had four or more antenatal care visits for their last pregnancy, and 67.1% delivered in a health institution. In contrast, 32.9% did not receive postnatal care. Regarding nutrition education, 71.6% of women reported receiving counseling from health professionals.

**TABLE 2:** Lactating women’s utilization of health services in Wondo Genet district of Sidama Region.

Variable (*n* = 401)	Category	Frequency	Percent (%)
Four or more ANC visits	Yes	324	80.8
	No	77	19.2

Place of delivery	Health institution	269	67.1
	Home	132	32.9

Postnatal care	Yes	269	67.1
	No	132	32.9

Nutrition counseling	Yes	287	71.6
	No	114	28.4

### 3.3. Knowledge and Consumption of Vitamin A‐Rich Foods

**FIGURE 1:**
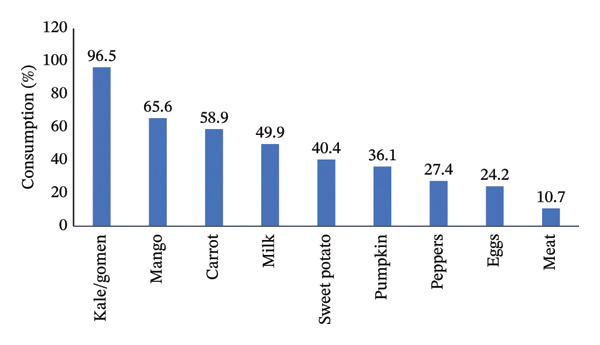
Vitamin A‐rich foods consumed by lactating women in Wondo Genet district, Sidama Region.

The study found that half of the respondents (50.1%) demonstrated good knowledge regarding vitamin A. The most commonly consumed vitamin A‐rich foods in the study area were Ethiopian kale (96.5%), mango (65.6%), carrot (58.9%), and milk (49.9%). In contrast, eggs (24.2%) and meat (10.7%) were the least frequently consumed sources of vitamin A (Figure 1).

### 3.4. Dietary Diversity and Adequacy of Dietary Vitamin A Intake of Lactating Women

**FIGURE 2:**
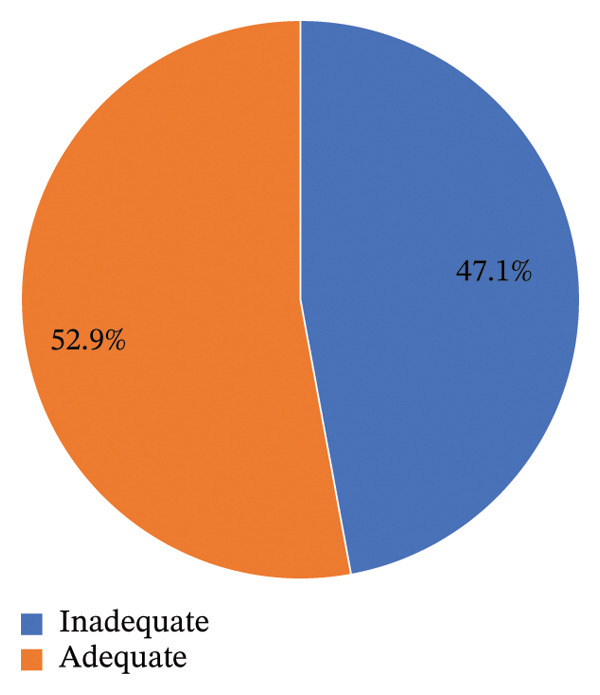
Dietary vitamin A intake adequacy among lactating women in Wondo Genet district, Sidama Region.

Cereals, roots, and leafy vegetables were the primary food sources in the study area, consumed by 99.5% and 87.9% of participants, respectively. The dietary diversity among lactating women was low, with a mean score of 4.14 (±1.53). Over two‐thirds (69.3%) consumed foods from fewer than 5 of the 10 key groups. Median vitamin A intake was 456.5 μg retinol activity equivalent (IQR: 412.6, 491.9). The prevalence of inadequate vitamin A intake was 47.1% (95% CI: 42.2, 52.0) (Figure 2). When assessed against the RDA, the inadequacy prevalence rose to 73.6% (95% CI: 69.2, 77.9).

### 3.5. Factors Associated With Inadequate Dietary Vitamin A Intake

Table 3 presents the crude and adjusted estimates for inadequate vitamin A intake. In the adjusted model, lactating women with five or more family members had 1.68‐fold increased odds of inadequately consuming dietary vitamin A compared to their counterparts (AOR = 1.68; 95% CI: 1.10, 2.58). Lactating women from food‐insecure households had 1.6 times higher odds of inadequate dietary vitamin A intake (AOR = 1.60; 95% CI: 1.05, 2.44). Study participants without nutrition counseling had about three times higher odds (AOR = 2.67; 95% CI: 1.66, 4.28) of inadequate dietary vitamin A intake compared to those who received counseling. Lactating women with low dietary diversity scores had 1.65 times higher odds (AOR = 1.65; 95% CI: 1.04, 2.62) of consuming inadequate dietary vitamin A compared to those with higher scores. Consuming fewer than three meals per day was associated with 2.18 times higher odds of inadequate dietary vitamin A intake (AOR = 2.18; 95% CI: 1.41, 3.37) compared to consuming three or more meals.

**TABLE 3:** Factors associated with dietary vitamin A intake inadequacy among lactating women in Wondo Genet district of Sidama Region.

Variable (*n* = 401)	Dietary vitamin A intake		COR (95% CI)	AOR (95% CI)	*p* value
	Inadequate	Adequate			
Husband education					
No formal education	63	57	1.36 (0.89, 2.09)	1.12 (0.63, 1.99)	0.695
Primary and above	126	155	1.00	1.00	
Husband occupation					
Farmer	94	100	1.11 (0.75, 1.64)	1.06 (0.69, 1.63)	0.779
Other works	95	112	1.00	1.00	
Family size					
≥ 5 members	99	88	1.55 (1.04, 2.30)	1.68 (1.10, 2.58)	< 0.001^∗^
< 5 members	90	124	1.00	1.00	
Food security					
Insecure	105	90	1.69 (1.14, 2.52)	1.60 (1.05, 2.44)	0.028^∗^
Secured	84	122	1.00	1.00	
Place of delivery					
Home	72	60	1.56 (1.02, 2.37)	1.68 (0.95, 2.98)	0.072
Health institution	117	152	1.00	1.00	
Received PNC					
No	122	127	1.22 (0.81, 1.83)	1.27 (0.82, 1.97)	0.282
Yes	67	85	1.00	1.00	
Received nutrition counseling					
No	73	41	2.62 (1.67, 4.11)	2.67 (1.66, 4.28)	< 0.001^∗^
Yes	116	171	1.00	1.00	
Vitamin A knowledge					
Poor	126	130	1.26 (0.84, 1.90)	1.22 (0.79, 1.89)	0.368
Good	63	82	1.00	1.00	
Dietary diversity score					
Low	112	141	0.73 (0.49, 1.10)	1.65 (1.04, 2.62)	0.031^∗^
High	77	71	1.00	1.00	
Meals consumed per day					
< 3 per day	91	63	2.19 (1.46, 3.31)	2.18 (1.41, 3.37)	< 0.001^∗^
≥ 3 per day	98	149	1.00	1.00	

*Note:* 1.00 represents a reference category.

^∗^ *p* value < 0.05.

## 4. Discussion

A community‐based cross‐sectional study was conducted in the Wondo Genet district of the Sidama Region to identify factors associated with dietary vitamin A intake among lactating women. The median dietary vitamin A intake was 456.5 µg retinol activity equivalents (IQR: 412.6‐491.9). Overall, 47.1% of the lactating women had inadequate dietary vitamin A intake. Family size, household food security status, nutrition counseling, dietary diversity, and meal frequency were identified as independent predictors of inadequate dietary vitamin A intake.

Our study presented the 47.1% prevalence of inadequate dietary vitamin intake among lactating women. A study conducted in Cameroon reported a lower prevalence (37.5%) [29]. Studies conducted in different parts of Ethiopia (58.2%‐86.7%) [15, 16, 30], Kenya (88%) [31], Bangladesh (87%) [32], and Brazil (74%) [33] reported even a higher prevalence of inadequate vitamin A intake among lactating women. The high dietary vitamin A inadequacy could be due to low nutrition knowledge among women, resulting in insufficient consumption of vitamin A‐rich foods (meat, fish, milk, eggs, vegetables, and fruits). Food insecurity and limited purchasing power of lower‐middle wealth families also hinder access to a diverse vitamin A‐rich diet.

The study found a significant association between receiving nutrition counseling during health institution visits and inadequate dietary vitamin A intake among lactating women. The odds of becoming dietary vitamin A inadequate were about three times higher in lactating women who did not receive nutrition counseling compared to their counterparts. Consistent findings were reported from central Ethiopia [34] and northeast Ethiopia [35]. The possible explanation for this result is that mothers without nutrition counseling may lack awareness of diverse food sources, leading to inadequate intake of vitamin A‐rich foods.

Our study found that lactating women from food‐insecure households had significantly higher odds of inadequate dietary vitamin A intake compared to those from food‐secure households. Moreover, the study showed that women from food insecure households had reduced consumption of vitamin A‐rich foods. This finding aligns with studies conducted in northern Ethiopia [30] and Kenya [36]. This could be due to the fact that household food insecurity impacted the availability, accessibility, and utilization of vitamin A‐rich food in lactating women [37].

A low dietary diversity score was significantly associated with inadequate dietary vitamin A intake. Lactating women consuming a less diversified diet had 1.65 times higher odds of inadequate vitamin A intake compared to those with a more diversified diet. This finding was in line with findings from other places such as northwest Ethiopia [16], northeast Ethiopia [15], and Indonesia [38]. Lactating mothers with low dietary diversity may consume inadequate vitamin A due to limited access to vitamin A‐rich foods (e.g., fruits, vegetables, and animal products), driven by poverty, food insecurity, or cultural restrictions [12]. Moreover, insufficient nutrition education and awareness about vitamin A’s importance can further contribute to poor intake [30].

The number of meals consumed per day showed a significant association with dietary vitamin A intake. Lactating women who consumed fewer than three meals per day had about two times higher odds of having inadequate dietary vitamin A intake compared to those who consumed three or more meals per day. This finding was in line with a study conducted northwest Ethiopia [16] and Kenya [39]. This may happen because a decreased number of meals consumed per day leads to the consumption of less varied food, potentially resulting in inadequate vitamin A intake.

In the study, statistically significant association was observed in family size and inadequate dietary vitamin A intake. With reference to women with fewer than five family members, the odds of inadequate dietary vitamin A intake were 1.68 times higher among women who had five or more family members. Consistent finding was reported from studies conducted in Dessie town, Ethiopia [16], and Pawie district, Ethiopia [40]. The possible explanation for this is that a large family strains resources, increasing food sharing and potentially compromising maternal nutrition, resulting in inadequate vitamin A intake.

### 4.1. Strength and Limitation

This study employed a community‐based design to assess dietary vitamin A intake and associated factors among breastfeeding women in the study area using a multiple 24 h recall method. The use of simple random sampling based on health post records might lead to selection bias, as health extension workers may not regularly or adequately document information about recently delivered mothers. Participants’ memory limitations and the tendency to provide socially acceptable responses could influence the findings. The use of a single 24 h recall does not fully capture usual dietary patterns. In addition, the food composition tables available in low‐income settings may not precisely represent the nutrient composition of locally consumed foods, which could affect nutrient intake estimates.

### 4.2. Conclusion and Recommendation

The study found a high level of inadequate dietary vitamin A intake among lactating women in the area. Independent predictors of inadequate vitamin A intake among lactating women in the Wondo Genet district included large family size, food insecurity, lack of nutritional counseling, dietary diversity score, and the number of meals consumed per day. We recommend implementing targeted nutritional counseling and education programs for lactating women, focusing on vitamin A awareness, meal planning, and strategies to address food insecurity, especially in larger families.

**Nomenclature**
ANC: Antenatal Care
AOR: Adjusted Odds Ratio
CI: Confidence Interval
CL: Confidence Level
COR: Crude Odds Ratio
DDS: Dietary Diversity Score
EAR: Estimated Average Requirement
EFCT: Ethiopian Food Composition Table
EMR: Estimated Mean Requirement
FAO: Food and Agriculture Organization
HFIAS: Household Food Insecurity Access Scale
IRB: Institutional Review Board
IQR: Interquartile Range
PNA: Postnatal Care
RDA: Recommended Daily Allowance
SD: Standard Deviation
WHO: World Health Organization

## Author Contributions

Study conceptualization: Amelo Bolka, Aregahegn Dona, Selamawit Sikuare.

Data curation: Amelo Bolka, Assefa Philipos.

Formal analysis: Amelo Bolka, Selamawit Sikuare.

Investigation: Selamawit Sikuare, Aregahegn Dona.

Methodology: Amelo Bolka, Assefa Philipos.

Funding acquisition: Selamawit Sikuare.

Software: Amelo Bolka, Aregahegn Dona, Selamawit Sikuare.

Supervision: Amelo Bolka, Assefa Philipos.

Validation: Amelo Bolka, Assefa Philipos.

Writing original draft: Selamawit Sikuare, Aregahegn Dona.

Review and editing: Amelo Bolka, Aregahegn Dona, Assefa Philipos, Selamawit Sikuare.

## Funding

The study was funded by the Wondo Genet District Health Office and the Yirgalem Hospital Medical College.

## Disclosure

A preprint has previously been published [41].

## Ethics Statement

Ethical clearance was obtained from the Institutional Review Board (IRB) of Yirgalem Hospital Medical College (Protocol Number YHMC/IRB001, Date 22/11/2023). The college issued an official letter to the Wondo Genet District Health Office, and concerned officials were informed about the study’s purpose. Informed written consent has been collected from each respondent in the study. The study participants’ information was kept confidential using pseudonymous codes.

## Conflicts of Interest

The authors declare no conflicts of interest.

## Data Availability

The datasets analyzed during the current study are not publicly available due to institutional regulation but are available from the corresponding author on reasonable request.
